# Left trisectionectomy combined with resection of the right hepatic vein and inferior vena cava after right hepatic vein embolization for advanced intrahepatic cholangiocarcinoma

**DOI:** 10.1186/s40792-019-0655-0

**Published:** 2019-06-18

**Authors:** Toshihiro Suzuki, Tomoki Ebata, Yukihiro Yokoyama, Takashi Mizuno, Tsuyoshi Igami, Junpei Yamaguchi, Shunsuke Onoe, Nobuyuki Watanabe, Masato Nagino

**Affiliations:** Division of Surgical Oncology, Department of Surgery, Nagoya University Graduate School of Medicine, 65 Tsurumai-cho, Showa-ku, Nagoya, 466-8550 Japan

**Keywords:** Right hepatic vein embolization, Left hepatic trisectionectomy, Intrahepatic cholangiocarcinoma

## Abstract

**Background:**

When the inferior right hepatic vein (IRHV) is present, left hepatic trisectionectomy with resection of the right hepatic vein (RHV) is theoretically possible without reconstruction of the RHV. We here report a successful case of this extended hepatectomy after RHV embolization for advanced intrahepatic cholangiocarcinoma.

**Case presentation:**

A 71-year-old man was admitted to our clinic with abdominal pain. Computed tomography showed a cholangiocarcinoma located at the caudate lobe that involved the inferior vena cava (IVC) and the roots of the three major hepatic veins. Portal vein embolization of the left and right anterior portal veins was performed. As the IRHV was present but thin, RHV was also embolized. Left hepatic trisectionectomy with resection of the involved IVC and RHV, preserving the IRHV, was done. The IVC was reconstructed with artificial graft. The patient was discharged on postoperative day 36.

**Conclusion:**

RHV embolization is useful in extended left trisectionectomy with resection of the RHV when the IRHV is present but thin.

## Background

When the inferior right hepatic vein (IRHV) is present, left hepatic trisectionectomy with resection of the right hepatic vein (RHV) is theoretically possible without reconstruction of the RHV. When the IRHV is “thin,” clinical utility of RHV embolization was reported [1, 2]. We here report a successful case of left hepatic trisectionectomy combined with resection of the RHV and inferior vena cava (IVC), preserving the IRHV, after RHV embolization for advanced intrahepatic cholangiocarcinoma.

## Case presentation

**Fig. 1:**
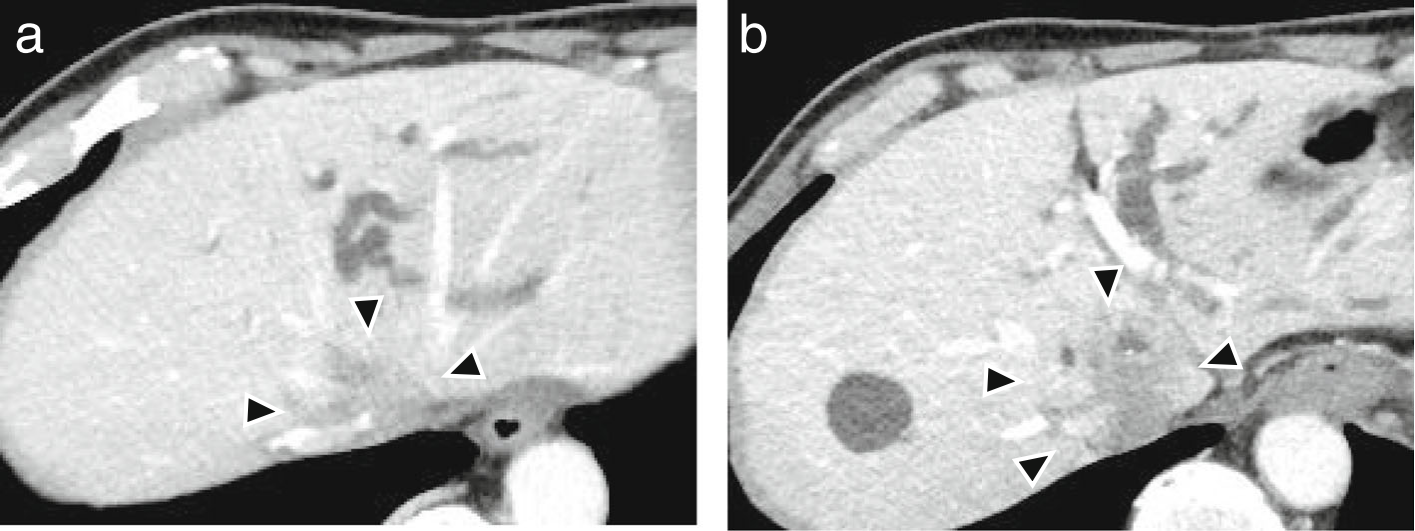
**a** Computed tomography showed an irregular tumor (arrowheads) involving the inferior vena cava and the root of the three major hepatic veins. **b** Left hepatic bile duct was dilated due to tumor invasion

A 71-year-old man was admitted to our clinic with abdominal pain. Contrast-enhanced computed tomography (CT) showed a tumor located at the caudate lobe that involved the IVC and the roots of the three major hepatic veins (Fig. 1a, b). The diagnosis of an advanced intrahepatic cholangiocarcinoma was made. Neither lymph node metastasis nor distant metastasis was detected. He had no jaundice and was in good general condition.

**Fig. 2:**
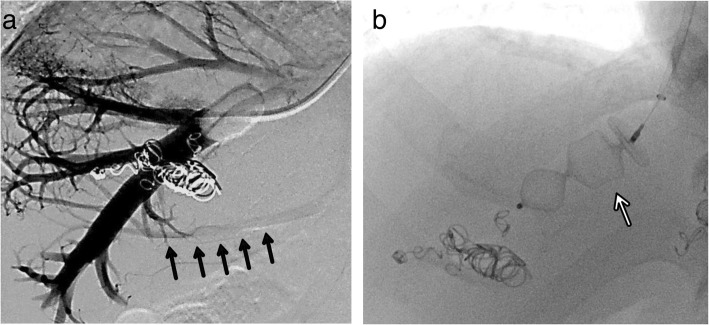
**a** Retrograde venography of the right hepatic vein under balloon occlusion showed a “thin” inferior right hepatic vein (arrows). **b** The right hepatic vein was embolized with an Amplatzer Vascular plug-II® (white arrow)

**Fig. 3:**
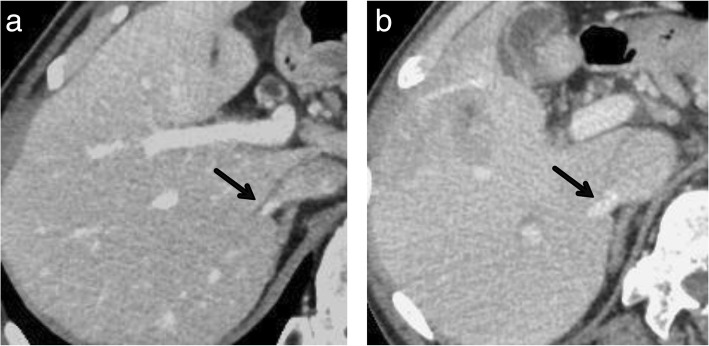
**a** Computed tomography obtained before embolization showed that the diameter of the inferior right hepatic vein (arrow) was 3.5 mm. **b** The diameter of the vein was enlarged to 7.7 mm 29 days after the right hepatic vein embolization

The only possible procedure to achieve curative resection was a left hepatic trisectionectomy combined with resection of the IVC and the three major hepatic veins. The volume of the right posterior sector was 333 cm^3^ (32.3% of the whole liver). The plasma disappearance rate of indocyanine green was 0.154. Portal vein embolization (PVE) of the left and right anterior portal veins was performed to increase the volume of the right posterior sector. In addition, as this case had a “thin” IRHV, embolization of the RHV was planned, with the aim of simplifying the surgical procedure by preserving the IRHV. Seven days after the PVE, the RHV was embolized using an Amplatzer vascular plug-II® (St. Jude Medical, St. Paul, Minnesota, USA), which was expected to develop collaterals from the RHV to the IRHV (Fig. 2a, b). To assess the feasibility of RHV resection, we ensured collaterals to the IRHV under balloon occlusion of the RHV. A CT scan obtained 29 days after the RHV embolization demonstrated that the volume of the right posterior sector had increased up to 562 cm^3^ (42.9% of the whole liver) and that the diameter of the IRHV had enlarged to 7.7 mm, from 3.5 mm before embolization (Fig. 3a, b).

**Fig. 4:**
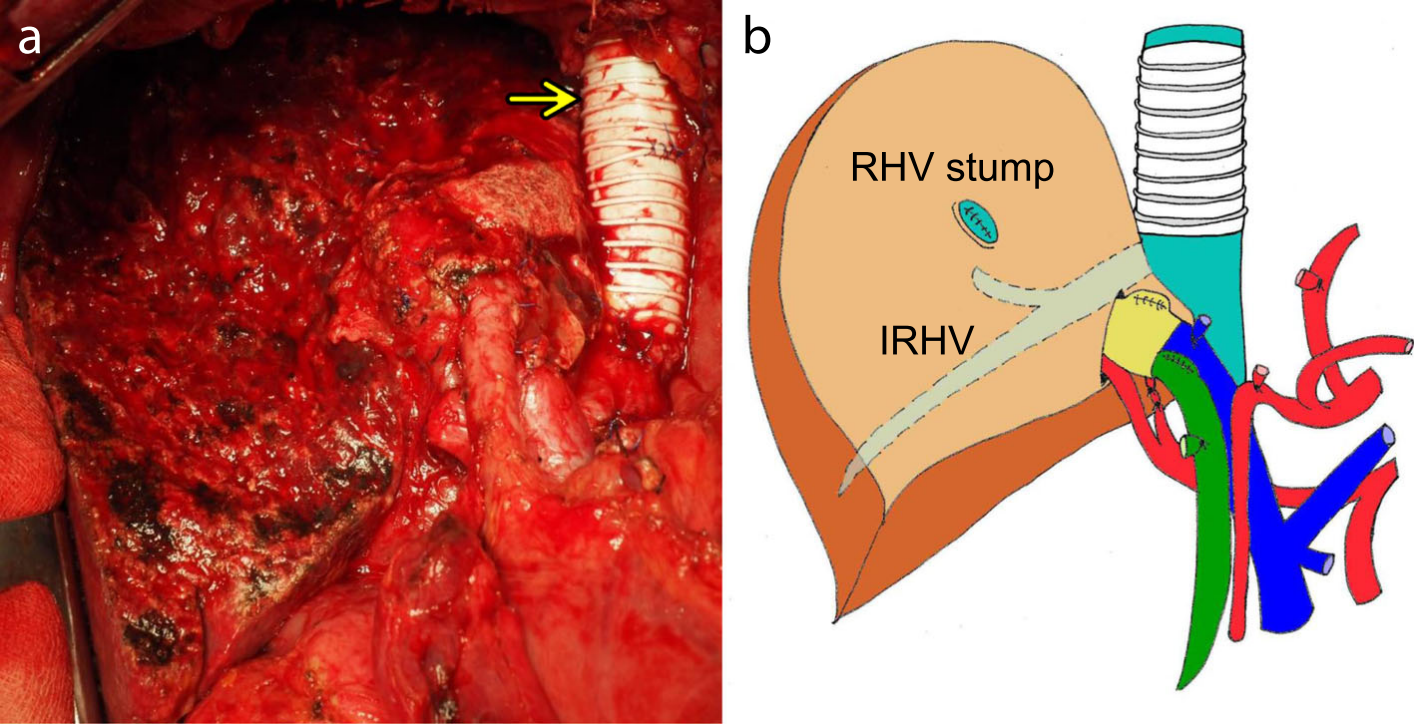
**a** Completion photograph after the left hepatic trisectionectomy combined with resection of the inferior vena cava and right hepatic vein. The inferior vena cava was reconstructed with an artificial graft (arrow). **b** Scheme of the operative view. RHV, right hepatic vein; IRHV, inferior right hepatic vein

Surgery was performed 35 days after the RHV embolization. A left hepatic trisectionectomy with partial resection of the caudate lobe was performed. The involved IVC and RHV were also resected *en bloc*, and the IRHV was preserved as planned. Before the involved IVC was resected, we placed the temporal venous-venous bypass between the IVC distal to the renal veins and the right atrium. The resected IVC was reconstructed using a polytetrafluoroethylene (PTFE) vascular graft (Fig. 4a, b). The operative time was 867 min, and blood loss was 12,428 mL.

**Fig. 5:**
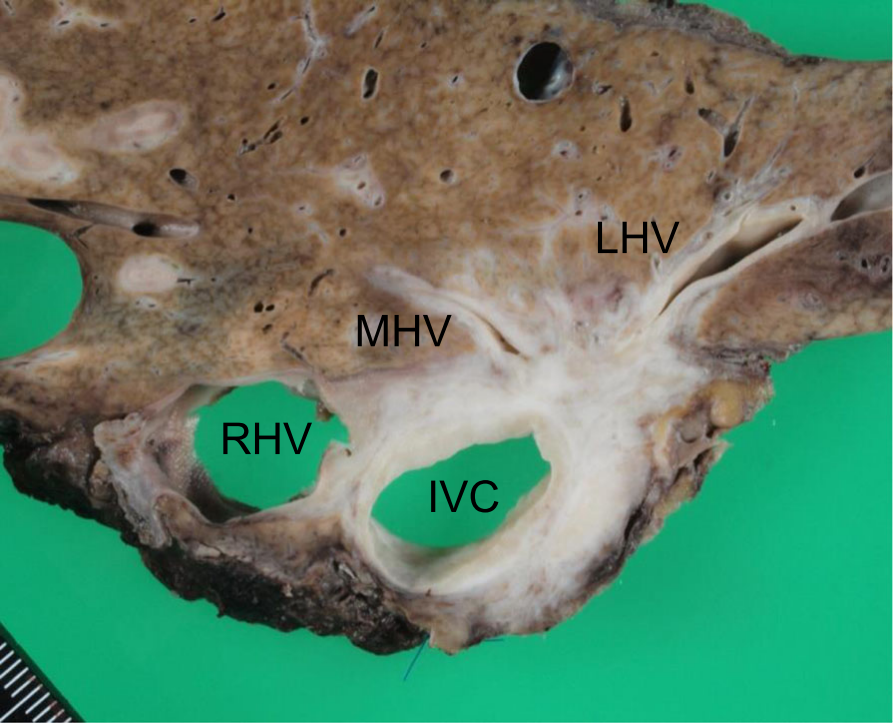
Cut surface of the resected specimen. RHV, right hepatic vein; MHV, middle hepatic vein; LHV, left hepatic vein; IVC, inferior vena cava

Histologically, the tumor was a moderately differentiated adenocarcinoma that had invaded the IVC and all three major hepatic veins and exhibited regional lymph node metastases (Fig. 5). Postoperatively, maximum serum total bilirubin concentration was 5.8 mg/dl (grade B liver failure). Mild ascites developed, but it was well controlled by diuretics. He was discharged from the hospital in good health on postoperative day 36 and enjoyed an active social life, but he died of recurrence 18 months after the surgery.

## Discussion

Hepatectomy combined with the simultaneous resection of the IVC and three major hepatic veins represents one of the most complicated and challenging procedures in hepatobiliary surgery. An “ex vivo” or “ante situm” technique is often required to resect the tumor followed by reconstruction of the IVC and one of the major hepatic veins. However, the presence of the IRHV can circumvent RHV reconstruction even in cases of such an extended hepatectomy. In 1987, Makuuchi et al. mentioned the theoretical feasibility of an IRHV-preserving left hepatic trisectionectomy with resection of the RHV [3]. Thereafter, several authors reported this type of extended hepatectomy in patients having the IRHV [4, 5]; in these reports, RHV embolization was unnecessary because the IRHV was originally “thick.” On the other hand, in patients with a “thin” IRHV like that in the present case, omitting reconstruction of the RHV may be risky due to possible congestion of the remnant liver. To avoid this potential risk, in 2003, one of the present authors (MN) introduced RHV embolization [1], which can help to develop collaterals from the RHV to the IRHV; in that previous case, the tumor involved the RHV but not the IVC; thus, the IVC was not resected. In the present case, the IVC was extensively involved, requiring IVC resection with reconstruction. If the IRHV had been absent, or if the RHV had not been embolized, a curative *en bloc* resection would have been very difficult.

After RHV embolization, we should wait at least 2 weeks to develop enough collaterals from the RHV to the IRHV. The previous experimental study reported that adequate intrahepatic collaterals develop rapidly after hepatic vein occlusion and are established within 2 weeks in large animals [6].

RHV embolization has a potential risk of coil migration leading to pulmonary embolism. To date, however, no serious procedure-related complications were reported [7, 8]. We thought that use of Amplatzer vascular plug could reduce the risk of such migration, providing the plug is oversized by at least 50%.

An important topic of discussion is the definition of “thin” or “thick,” which is vague and based on an empirical impression. In other words, to what extent does the diameter of the IRHV guarantee the safety of this extended hepatectomy? Although this issue is still unclear, one previous study classified a large-caliber IRHV as a vein with a diameter of > 18 mm and a medium-sized IRHV as a vein with a diameter of 5 to 18 mm [9]. Accordingly, the IRHV in the present case is apparently defined as “thin.”

## Conclusions

When a left hepatic trisectionectomy combined with the IVC and the RHV resection is planned, surgeons should focus on the presence or absence of the IRHV and, if it is present but thin, should consider RHV embolization before surgery.

## Abbreviations

CT: Computed tomography
IRHV: Inferior right hepatic vein
IVC: Inferior vena cava
PTFE: Polytetrafluoroethylene
PVE: Portal vein embolization
RHV: Right hepatic vein
